# Ammonium Bisulfite and Urea–Metabisulfite as Formaldehyde Scavengers in Low-Molar-Ratio Urea–Formaldehyde Resin for Medium-Density Fiberboard: Curing Behavior and Panel Performance

**DOI:** 10.3390/polym18070786

**Published:** 2026-03-25

**Authors:** Viktoria Dudeva, Georgi Ivanov, Viktor Savov, Petar Antov, Konstantinos Ninikas, Stoyko Petrin, Alexandrina Kostadinova-Slaveva

**Affiliations:** 1 Faculty of Forest Industry, University of Forestry, 1797 Sofia, Bulgaria; v.dudeva@ltu.bg (V.D.); georgi_ivanov@ltu.bg (G.I.); 2 Center of Competence “Clean Technologies for a Sustainable Environment—Water, Waste, Energy for a Circular Economy”, 1407 Sofia, Bulgaria; aslaveva@ltu.bg; 3 Department of Forestry, Wood Sciences & Design, School of Technology, University of Thessaly, 43100 Karditsa, Greece; kninikas@uth.gr; 4 Faculty of Chemical Technologies, University of Chemical Technology and Metallurgy, 1757 Sofia, Bulgaria; stpetrin@uctm.edu; 5 Faculty of Ecology and Landscape Architecture, University of Forestry, 1797 Sofia, Bulgaria

**Keywords:** medium-density fiberboard, urea–formaldehyde resin, low molar ratio, ammonium bisulfite, urea–metabisulfite, formaldehyde scavenger, perforator method, curing behavior, simultaneous thermal analysis

## Abstract

Ultra-low-formaldehyde medium-density fiberboard (MDF) is commonly produced using low-molar-ratio urea-formaldehyde (UF) resins; however, the reduced formaldehyde-to-urea ratio also lowers resin reactivity and can complicate curing. The aim of this research work was to investigate and evaluate the performance of ammonium bisulfite and urea–metabisulfite as formaldehyde scavengers for a low-molar-ratio UF resin (F/U = 1.06) at 1, 3, and 5 wt% (based on dry UF resin solids) used for MDF panel manufacturing. The modified adhesive systems were first screened by simultaneous thermal analysis in air to determine changes in the curing profile, and laboratory panels were then produced and evaluated for formaldehyde content by the perforator method (EN ISO 12460-5:2015) and for the main physical and mechanical properties. Ammonium bisulfite shifted the main curing peak to higher temperatures, indicating stronger retardation of the principal polycondensation stage, whereas urea–metabisulfite generated a broader, multi-peak curing profile. Despite these differences, both additives reduced the perforator values substantially. The control MDF already met the E0 level (3.84 mg/100 g oven-dry board), while 3 wt% ammonium bisulfite and 5 wt% urea–metabisulfite reached the super E0 levels (<1.5 mg/100 g; 1.36 and 1.26 mg/100 g, respectively). To note, scavenger addition up to 5 wt% (based on dry UF resin solids) did not significantly affect density, water absorption/thickness swelling, or bending and internal bond properties. The results demonstrate that sulfite-based scavengers can be incorporated into low-molar-ratio UF adhesives to obtain ultra-low-formaldehyde MDF while maintaining the main panel properties.

## 1. Introduction

Wood-based panels, particularly medium-density fiberboard (MDF), are among the most important engineered lignocellulosic products for furniture and interior applications. Their industrial success is closely related to the extensive use of urea–formaldehyde (UF) resins, which combine high reactivity, good adhesion to wood, relatively low curing temperature, short press times, water solubility, and low cost [1,2]. Nevertheless, UF-bonded panels remain under continuous regulatory and scientific scrutiny because residual (free) formaldehyde in the adhesive and formaldehyde released from finished boards represent key environmental and health-related concerns [1,2,3].

In order to meet increasingly stringent limits for formaldehyde release, a widely adopted approach is to decrease the formaldehyde-to-urea (F/U) molar ratio of UF resins [3,4,5,6,7,8,9,10]. Although this approach effectively lowers the formaldehyde release potential of wood composites, it also reduces resin reactivity and can narrow the processing window in industrial pressing [6,7,8,11,12,13,14,15,16,17,18,19,20]. In low-molar-ratio UF adhesive systems, the reduced abundance of reactive methylol species and condensation products can slow network formation and increase sensitivity to formulation variables such as catalyst type, pH, moisture content, and the presence of additives. As a result, the targeted reduction in formaldehyde release is often accompanied by a risk of delayed cure, incomplete crosslinking, or compromised board properties if the formulation is not carefully balanced [6,7,8,9,10,11,12].

Because of this compromise between lower formaldehyde release and reduced resin reactivity, formaldehyde scavengers are frequently employed as a complementary route to produce low-formaldehyde wood-based panels [3,21,22,23,24,25,26,27,28,29,30,31,32,33]. A broad range of scavengers has been explored, including urea and other amines, tannins, mineral fillers, nanoparticles, and sulfite-based compounds [3,21,25,26,27,34,35,36,37]. Sulfite and metabisulfite systems are particularly attractive because they can react with formaldehyde to form addition products, thereby reducing the amount of unbound formaldehyde available for subsequent release [21,22]. However, scavenger performance should not be assessed solely by reductions in measured formaldehyde values because scavengers may also affect resin pH, cure kinetics, rheology, hydrolytic stability, and, ultimately, the physical and mechanical properties of the panels [3,17,18,19].

Previous works on formaldehyde scavengers in wood-based panels have shown that sodium metabisulfite and ammonium bisulfite can substantially reduce formaldehyde release, but their overall effectiveness depends on the scavenger form, its distribution within the panel structure, and the testing method used to quantify formaldehyde [21,22]. Costa et al. reported that sodium metabisulfite, particularly in solid form, can provide strong reductions, while ammonium bisulfite also exhibits high scavenging potential but may impose a more pronounced penalty in internal bond strength and thickness swelling under some conditions [21]. The same study highlighted that scavenger performance can differ markedly among perforator, desiccator, and gas analysis methods, consistent with differences in the stability and reversibility of formaldehyde adducts under varying temperature and humidity conditions [21,24]. In parallel, review studies have emphasized that urea-based scavengers are inexpensive and reactive toward formaldehyde, but they may also decrease resin reactivity and, in some cases, impair bonding or lose effectiveness under humid conditions due to hydrolysis of formed species [3,25].

From a polymer-science perspective, the central challenge is therefore not only to capture formaldehyde but to preserve a curing pathway compatible with hot pressing and final board performance. This issue is especially critical for low-molar-ratio UF resins, whose curing behavior is inherently more sensitive than that of conventional UF systems [6,7,8,9,10,11,12]. Prior work on low-molar-ratio UF resins has demonstrated that relatively small formulation changes can significantly influence condensation kinetics, cure evolution, and adhesive performance in such systems [6,7,8,9,10,11,12,13]. Additive-modified UF resins may thus exhibit altered thermal behavior, delayed cure, broadened curing intervals, and changes in the balance between early-stage reactions and the main polycondensation step [6,11,12,13,14,15,16]. In this context, thermal analysis is not merely a supplementary characterization tool, but an important method for linking resin modification, processing behavior, and panel properties [11,12,13,14,15,16].

Despite the extensive literature on low-formaldehyde adhesives and scavengers, an important research gap remains. Most published studies focus either on general reviews of scavenger classes, on particleboard rather than MDF, or on formaldehyde emission reduction, without a sufficiently detailed discussion of curing behavior in low-molar-ratio UF systems [3,21,25,38,39,40]. While scavenger-modified UF systems and their curing behavior have been investigated, direct comparisons between ammonium bisulfite and urea-metabisulfite in low-molar-ratio UF resin formulations for MDF are still limited [15,16]. Even fewer studies combine resin-level thermal analysis with board-scale validation through standardized formaldehyde testing and evaluation of key physical and mechanical properties. This lack of integrated evidence complicates the assessment of whether a scavenger merely lowers measured formaldehyde values or can be considered technologically suitable for producing ultra-low-formaldehyde MDF panels with retained performance.

Accordingly, the present study addresses the insufficiently clarified combined effects of sulfite-based scavengers on curing behavior, formaldehyde reduction efficiency, and MDF performance when using a low-molar-ratio UF resin. A scavenger that lowers formaldehyde values may simultaneously retard cure, shift the effective curing interval to higher temperatures, or negatively affect bonding quality and dimensional stability of the resulting panels [3,15,17,21]. Therefore, an appropriate comparative assessment must consider not only formaldehyde reduction but also adhesive cure development and the resulting MDF panel properties.

Therefore, the aim of the present research work was to comparatively evaluate ammonium bisulfite and urea–metabisulfite as formaldehyde scavengers in a low-molar-ratio UF resin for MDF manufacturing. The study was designed to link resin-level behavior to panel-level performance by characterizing the curing features of the modified adhesive systems using simultaneous thermal analysis, and producing laboratory MDF panels for subsequent formaldehyde testing and selected physical and mechanical property evaluation. In doing so, the work clarifies which scavenger system provides the more favorable balance between reduced formaldehyde values, acceptable curing behavior, and retained MDF panel performance.

## 2. Materials and Methods

### 2.1. Raw Materials and Adhesive Systems

Industrial wood fibers for medium-density fiberboard (MDF) manufacturing and a low-molar-ratio urea-formaldehyde (UF) resin were supplied by Kronospan Bulgaria, Burgas, Bulgaria. The UF resin had a solids content of 50% and a formaldehyde-to-urea molar ratio of 1.06. The formaldehyde scavengers investigated in this work were ammonium bisulfite, supplied as the commercial product ALTON CAPTOR 315 (WIZ Chemicals, Dairago, Italy), and urea–metabisulfite, supplied as the commercial product White Solution (Kastamonu Entegre, Stara Zagora, Bulgaria). Ammonium sulfate was used as the hardener at 2 wt% (based on oven-dry UF resin solids), and a paraffin emulsion was added as a hydrophobizing agent at 1 wt% (based on oven-dry fibers). The scavenger loadings in all modified systems were expressed as wt% relative to oven-dry UF resin solids.

Two series of modified adhesive systems were prepared. In the first group, ammonium bisulfite was incorporated into the UF resin at 1, 3, and 5 wt%. In the second series, urea–metabisulfite was incorporated at the same loading levels. The unmodified UF resin served as the reference system. In total, seven adhesive formulations were investigated: control UF; UF with 1, 3, and 5 wt% ammonium bisulfite; and UF with 1, 3, and 5 wt% urea–metabisulfite.

### 2.2. Thermal Analysis of the Adhesive Systems

The curing-related thermal behavior of the adhesive systems was evaluated prior to panel manufacturing by simultaneous thermal analysis using an HS-TGA-103 thermogravimetric analyzer (Yantai Stark Instrument Co., Ltd., Yantai, China) equipped with heat-flow registration. Measurements were performed in an air atmosphere using open platinum crucibles. Samples were heated from room temperature to 220 °C at a heating rate of 5 °C min^−1^. This experimental protocol was adopted as a comparative screening regime to reveal formulation-dependent shifts in the curing interval and changes in the thermal profile of modified low-molar-ratio UF systems [6,11,12,13,14,15,16].

For each formulation, the onset temperature of the main exothermic event, peak temperature, and end temperature were determined from the corrected heat-flow curve. In addition, peak amplitude, normalized heat-flow intensity, apparent thermal effect, mass loss up to the final temperature, and residual mass at the principal peak temperature were recorded. In the case of broadened or multi-step curing profiles, the principal peak temperature was assigned to the most intense exothermic minimum of the corrected curve, while the overall shape of the exotherm was considered in the interpretation of the curing process. Because the measurements were performed in air and in open crucibles, the calculated thermal effect was used as a comparative parameter between formulations rather than as an absolute reaction enthalpy [13,14,15,16].

Viscosity and gelation time were not determined because they depend strongly on temperature, hardener/catalyst addition, and the measurement protocol. Instead, curing behavior was compared under identical screening conditions (air, open crucibles) using Tonset, Tpeak, Tend, curing interval, and normalized heat-flow intensity as the primary descriptors.

Thermal analysis was used as a preliminary screening tool for selecting the most promising scavenger loadings and for clarifying whether the additives shifted, broadened, or redistributed the principal curing stage of the low-molar-ratio UF resin. This step was considered essential because low-molar-ratio UF systems are known to be less reactive than conventional UF resins, and formaldehyde scavengers can further modify the curing pathway, rheology, and thermal stability of the adhesive [6,7,8,15,17,18,19,41,42,43].

### 2.3. Manufacture of Laboratory MDF Panels

Based on the thermal screening, laboratory MDF panels were manufactured with a nominal thickness of 10 mm and a target density of 760 kg m^−3^. The resin content was 8 wt% dry UF resin solids, based on oven-dry fibers. For the modified panels, the scavenger content was set at 1, 3, or 5 wt% based on dry resin solids. Ammonium sulfate hardener was added at 2 wt% based on dry resin solids, and paraffin emulsion was added at 1 wt% based on dry fibers.

The fibers were blended with the adhesive formulations until a uniform resin distribution was achieved, after which mats were manually formed and pre-pressed. Hot pressing was performed in a laboratory press (Manni PMC ST 100, Manni Srl, Campione, Italy) at 180 °C. The press time was determined using a pressing factor of 30 s mm^−1^, giving a total press time of 300 s for 10 mm panels. This regime was selected as a conservative curing schedule for low-molar-ratio UF-bonded MDF, taking into account the potentially retarding effect of sulfite-based scavengers on the main curing stage. Compared with lower-density lignocellulosic panels, where shorter pressing factors are often sufficient, the higher density of MDF and the chemically modified UF systems investigated here required an increased curing safety margin to promote reliable through-thickness hardening and to improve comparability among panel series [2,44,45].

### 2.4. Conditioning and Testing of the MDF Panels

After hot pressing, the laboratory panels were conditioned under standard climatic conditions of 20 ± 2 °C and 65 ± 5% relative humidity until constant mass. The panels were then cut into test specimens according to the relevant European standards.

The apparent density was determined according to EN 323 [46]. Thickness swelling after water immersion was determined according to EN 317 [47]. The modulus of rupture (MOR) and modulus of elasticity (MOE) in static bending were determined according to EN 310 [48]. Internal bond strength was determined as tensile strength perpendicular to the plane of the board according to EN 319 [49], using a WDW-50E universal testing machine (HST, Jinan, China) equipped with the appropriate fixtures for wood-based panel testing. For each MDF formulation, eight specimens (*n* = 8) were tested for each physical and mechanical property.

Formaldehyde content was determined by the perforator method according to EN ISO 12460-5:2015 [50]. This method was selected deliberately because the aim of the present study was to compare the formaldehyde potential of panels manufactured with different scavenger-modified UF adhesive systems under a single, well-established analytical framework. Because the apparent efficiency of formaldehyde scavengers can differ substantially among perforator, desiccator, and gas analysis methods owing to method-dependent temperature, humidity, and air-exchange conditions that affect the stability of formaldehyde–scavenger adducts [21,24], the perforator value was used as the primary comparative indicator in the present stage of the study.

### 2.5. Experimental Design and Data Evaluation

The experimental design included one control MDF panel series and six modified series, corresponding to the two scavenger types and three loading levels (1, 3, and 5 wt%). The results of the thermal analysis and panel testing were evaluated comparatively in order to identify the relationships between scavenger chemistry, curing behavior, formaldehyde reduction, and selected physical and mechanical properties. The data were expressed as mean values with the corresponding measures of dispersion, and the significance of the observed differences between formulations was evaluated statistically.

## 3. Results and Discussion

### 3.1. Thermal Analysis of the UF Adhesive Systems Modified with Ammonium Bisulfite and Urea–Metabisulfite

Thermal screening showed that the two sulfite-based formaldehyde scavengers affected the low-molar-ratio UF resin in markedly different ways. The unmodified resin exhibited a relatively well-defined main exothermic curing event with a peak temperature of 124.45 °C, which was used as the reference for the comparative interpretation of the modified systems. In contrast, the scavenger-containing formulations showed either a systematic shift in the main curing peak toward higher temperatures or a redistribution of the exothermic process into broader and partially overlapping thermal events. This distinction is technologically important because low-molar-ratio UF resins already operate at lower reactivity than conventional UF systems, and additional scavenger-induced retardation may further narrow the processing window during hot-processing [6,7,8,11]. From a processing perspective, a peak shift to higher temperature implies that a greater fraction of the main polycondensation may occur later in the press cycle, potentially increasing the risk of incomplete curing through the panel thickness under standard schedules. Conversely, a broadened or multi-step exotherm suggests a more complex cure development in which early-stage reactions and the principal network-forming step may partially decouple, which can affect both heat release dynamics and the development of bond strength [12,16].

**Figure 1:**
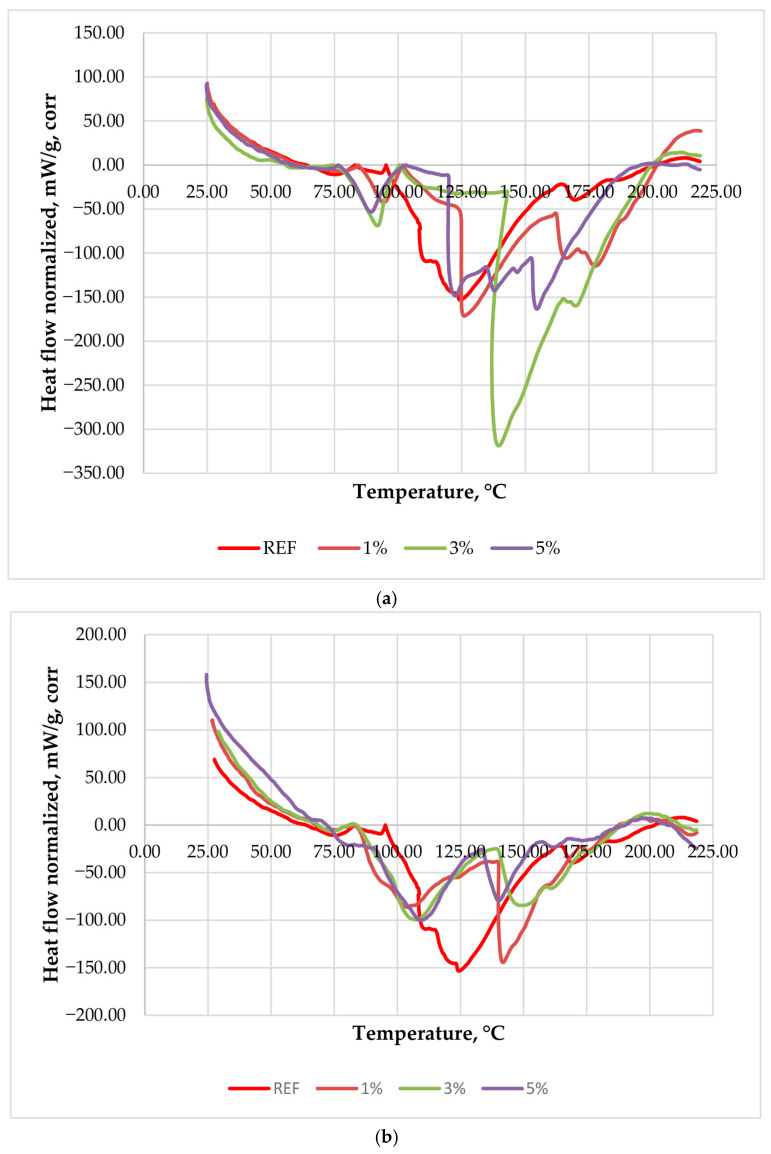
Baseline-corrected and mass-normalized heat-flow curves of the low-molar-ratio UF resin modified with (**a**) ammonium bisulfite and (**b**) urea–metabisulfite at 1, 3, and 5 wt%, compared with the reference resin (REF). Exothermic effects are plotted downward.

The corrected heat-flow curves of the control resin and the scavenger-modified systems are presented in Figure 1.

The figure clearly illustrates that the control UF resin shows a comparatively compact exothermic peak, whereas the modified systems exhibit either an upward shift of the main peak or a broadened and multi-step exothermic profile, depending on scavenger type and loading.

**Figure 2:**
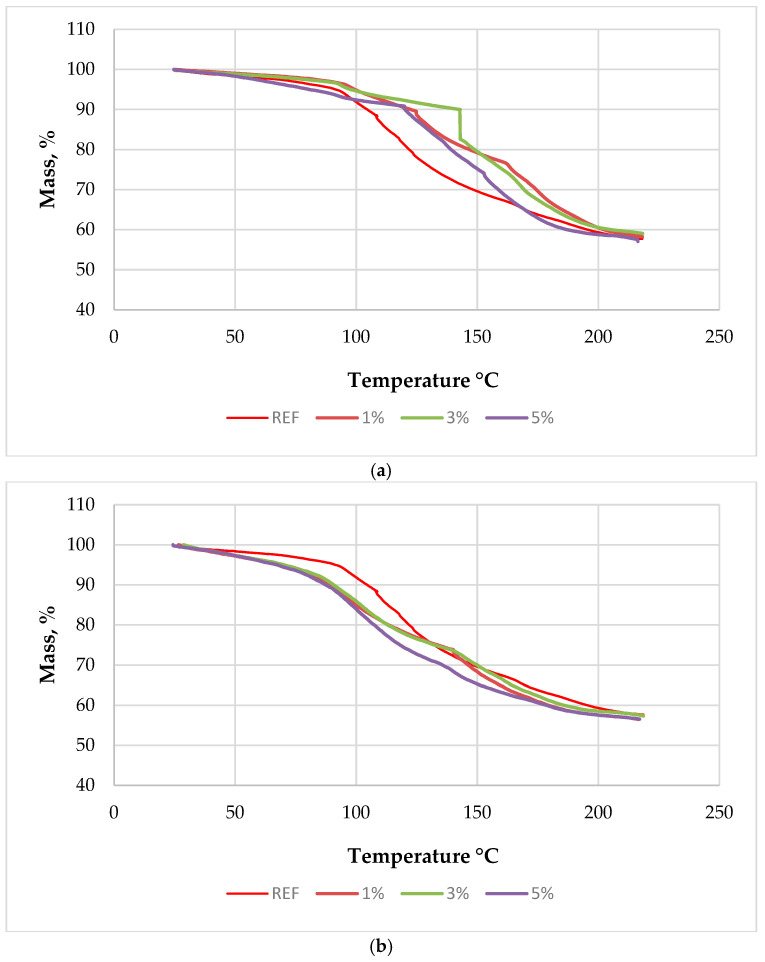
Mass-loss (TGA) curves of the low-molar-ratio UF resin modified with (**a**) ammonium bisulfite and (**b**) urea–metabisulfite at 1, 3, and 5 wt% (based on dry UF resin solids), compared with the reference resin (REF). Measurements were performed in air at a heating rate of 5 °C min^−1^ using open crucibles.

To support the interpretation of the curing-related exotherms, the corresponding mass-loss (TGA) curves are shown in Figure 2.

Over the investigated temperature range (up to 220 °C), all formulations exhibited a broadly comparable mass-loss trend, dominated by evaporation of residual moisture and low-molecular volatiles, without an abrupt early mass-loss step indicative of thermal degradation. Therefore, the scavenger-dependent differences observed in the corrected heat-flow curves can be attributed primarily to changes in cure development rather than to markedly different volatilization behavior among the formulations. From a processing perspective, the upward shift of the principal exothermic event in the ammonium bisulfite series implies that a larger fraction of the main UF polycondensation and network formation occurs at higher temperature, i.e., later within a fixed heating/pressing profile, which may require additional thermal reserve to ensure complete through-thickness curing. At 5 wt% ammonium bisulfite, the appearance of two partially separated exothermic maxima suggests partial separation of the curing process into overlapping stages, consistent with a stronger scavenging effect that redistributes the condensation reactions and decouples earlier structural rearrangements from the later formation of the main crosslinked network.

The pH measurements show that the two scavengers modified the UF resin in opposite directions. Ammonium bisulfite caused a gradual decrease in pH from 7.0 for the reference resin to 6.7, 6.5, and 6.0 at 1, 3, and 5 wt%, respectively. In contrast, urea–metabisulfite increased the pH to 7.5, 8.0, and 8.5 at the same loading levels. This opposite pH shift provides additional support for the different thermal curing profiles observed in the two systems. In the ammonium bisulfite series, the moderate decrease in pH may partially favor acid-catalyzed condensation. Still, this effect was evidently outweighed by the sequestration of formaldehyde, resulting in delayed curing at higher temperatures. In contrast, the progressive alkalization caused by urea–metabisulfite is consistent with the broader and more redistributed curing profile, since less acidic conditions are expected to reduce the rate and synchronization of UF polycondensation.

For the ammonium bisulfite series, the main curing peak moved from 124.45 °C for the control resin to 126.11, 139.49, and 154.52 at 1, 3, and 5 wt%, respectively (Table 1). Thus, the overall tendency was clear displacement of the principal exothermic event toward higher temperatures, especially at 3–5 wt%, indicating retardation of the dominant polycondensation stage. The 5 wt% ammonium bisulfite formulation showed a particularly interesting curing profile, characterized by the appearance of two partially separated exothermic maxima. This behavior suggests that, at this scavenger level, the curing of the low-molar-ratio UF resin no longer proceeds as a single dominant event, but rather as a two-step process. A plausible explanation is that the higher ammonium bisulfite content reduces the availability of free formaldehyde through bisulfite addition, thereby redistributing the condensation reactions and separating an earlier stage of structural rearrangement from the later formation of the main crosslinked UF network. Thus, the 5 wt% system may be interpreted as an intermediate case between a compact single-peak cure and a more strongly retarded curing process. The normalized peak intensity and apparent thermal effect were also generally higher for the ammonium bisulfite formulations, especially at 3 wt%, which suggests a more concentrated but thermally delayed main curing event.

This behavior is consistent with the known chemistry of bisulfite-based formaldehyde scavenging. Bisulfite ions react with formaldehyde to form hydroxymethanesulfonate-type adducts, thereby reducing the amount of freely available formaldehyde participating in methylolation and subsequent condensation reactions [3,21,22,25]. Since UF curing depends strongly on the availability of reactive methylol species and on the progress of acid-catalyzed polycondensation, partial sequestration of formaldehyde can delay the formation of the main crosslinked network [5,6,15,21]. In this sense, ammonium bisulfite appears to act mainly by preserving a relatively distinct principal cure peak while shifting it into a temperature region less favorable for easy MDF pressing. Similar trade-offs between effective formaldehyde reduction and impaired technological performance have been reported for bisulfite-type scavengers in wood-based panels [21,25].

The urea–metabisulfite formulations exhibited a distinctly different thermal pattern. The 1 wt% system showed a main peak at 141.84 °C, i.e., clearly higher than the control, but the formulations containing 3 and 5 wt% showed the deepest corrected minima at 107.79 and 108.87, respectively. If these peak temperatures were considered alone, one might conclude that the higher-content urea–metabisulfite systems cured earlier than the control resin. However, such an interpretation would be incomplete. In all urea–metabisulfite systems, and especially at 3–5 wt%, the exothermic region was broader and clearly multi-peak, with secondary shoulders or additional thermal events extending into the higher-temperature region. Therefore, the lower principal T_peak values should not be interpreted as a simple acceleration of curing, but rather as evidence of a redistributed and less synchronized curing process extending over a wider temperature interval. Although the 1 wt% urea–metabisulfite formulation exhibited a comparatively high principal peak temperature, it can still be regarded as part of the same overall curing trend. The difference is that at low dosage, the process remains more compact, while at higher dosages, the exothermic region becomes increasingly broadened and redistributed into partially overlapping stages [3,15].

A plausible explanation is that the urea–metabisulfite system introduces several concurrent or partially overlapping processes. Metabisulfite-derived species can capture formaldehyde through bisulfite-type addition reactions, while urea itself may also react with free formaldehyde to form methylolurea-type intermediates [3,21,22,25]. At the same time, urea-based scavengers are known to influence resin pH, alter the formaldehyde-to-urea balance, and often reduce the effective reactivity of UF adhesive systems [3,15,25,26,36]. In the present study, the observed multi-peak behavior most likely reflects a combination of formaldehyde capture, redistribution of reactive intermediates, and staged UF polycondensation rather than a single well-defined curing event. This interpretation agrees with previous reports that urea-containing scavenger systems can effectively lower formaldehyde but may also broaden the curing interval and affect the final adhesive performance [3,15,25].

The behavior of the 1 wt% urea–metabisulfite formulation deserves special attention. Unlike the higher concentrations, it showed a relatively high principal peak at 141.84 °C, which suggests that at low dosage, the additive was insufficient to reorganize the curing pathway into the more distributed multi-step pattern observed at 3–5 wt%. In other words, the system still behaved closer to a single dominant curing event, albeit shifted toward higher temperature. At higher scavenger loadings, the curing profile split into an earlier main minimum and later secondary events, indicating that the reaction pathway became more heterogeneous.

Overall, the thermal screening demonstrated that ammonium bisulfite acts mainly by shifting the principal UF curing event toward higher temperatures, whereas urea–metabisulfite causes a broader and multi-step curing process. The former effect is easier to interpret but clearly indicates cure retardation at higher loading levels; the latter appears less retarding if only the deepest minimum is considered, but in fact reflects a more complex and technologically less predictable cure profile. Therefore, the practically relevant scavenger range for subsequent MDF manufacture was considered to lie at or below 5 wt%, with 3 wt% ammonium bisulfite and 5 wt% urea–metabisulfite emerging as the most rational upper levels for further board validation.

### 3.2. Physical and Mechanical Properties of the MDF Panels

The results of the physical and mechanical tests show that the incorporation of ammonium bisulfite and urea–metabisulfite at levels of 1, 3, and 5 wt% did not cause substantial changes in the service-related properties of the laboratory-fabricated MDF panels. Across all panel series, the measured values remained within relatively narrow ranges, and one-way ANOVA confirmed that the observed differences were not statistically significant (*p* < 0.05). These results suggest that, under the selected manufacturing conditions, UF systems modified with sulfite-based scavengers can maintain panel performance comparable to the control UF resin. This outcome is technologically relevant because it indicates that formaldehyde-related parameters can be reduced without a measurable decrease in key physical and mechanical properties. Similar dose- and processing-dependent effects of formaldehyde scavengers on board performance have been reported previously, highlighting the importance of scavenger chemistry, loading level, and pressing regime [21,25,26,43].

#### 3.2.1. Density

**Figure 3:**
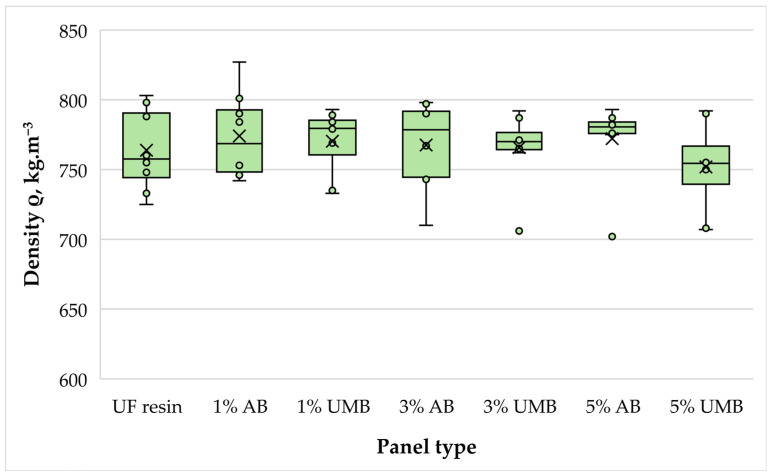
Density of the laboratory-fabricated MDF panels bonded with low-molar-ratio UF resin modified with ammonium bisulfite (AB) and urea–metabisulfite (UMB) at 1, 3, and 5 wt%.

The density of the produced MDF panels remained close to the target level and varied only within a comparatively narrow interval, from about 752 to 774 kg m^−3^ when group means are considered (Figure 3). The control panels had an average density of 763.8 kg m^−3^, while the modified series ranged from 751.9 kg m^−3^ (5 wt% urea-metabisulfite) to 774.0 kg m^−3^ (1 wt% ammonium bisulfite). One-way ANOVA showed that these differences were not statistically significant (*p* = 0.798), indicating that the addition of the investigated scavengers did not alter panel densification in a systematic manner. This is an important observation because it confirms that the differences observed in other properties cannot be attributed to density variation but should instead be interpreted in relation to the adhesive formulation itself. The consistent densities are also in line with the identical mat forming and pressing conditions applied across all series, supporting good repeatability of the laboratory process. Similar observations have been reported in the literature, where density typically remains stable at moderate scavenger dosages, and more pronounced changes are usually linked to major formulation shifts or modified pressing regimes [26,43].

#### 3.2.2. Water Absorption

**Figure 4:**
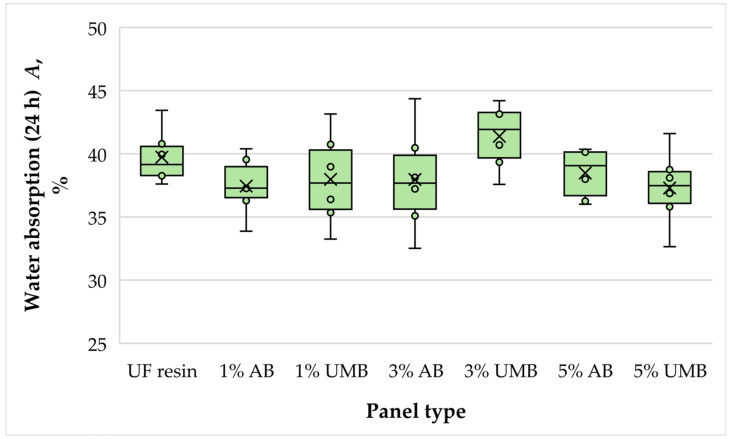
Water absorption of the laboratory-fabricated MDF panels bonded with low-molar-ratio UF resin modified with ammonium bisulfite (AB) and urea–metabisulfite (UMB) at 1, 3, and 5 wt%.

The mean water absorption (WA) values after immersion ranged from approximately 37.3% to 41.4% (Figure 4). The control panels exhibited an average value of 39.74%, and the scavenger-modified variants remained close to this level, with the lowest mean observed for the 5 wt% urea–metabisulfite panels (37.3%) and the highest for the 3 wt% urea–metabisulfite panels (41.38%). One-way ANOVA indicated that these differences were not statistically significant (*p* = 0.213). Therefore, within the investigated loading range, the incorporation of ammonium bisulfite or urea–metabisulfite did not measurably affect the water uptake of the MDF panels. This outcome suggests that, under the selected hot-pressing schedule, cure development and network formation were sufficient to maintain the moisture-response behavior of the panels despite the modified adhesive chemistry. In line with previous reports, WA is often among the first properties to respond to incomplete or non-uniform cure, but such effects are typically more evident at higher scavenger dosages and/or under less favorable pressing conditions [7,11,13]. The absence of a clear trend with scavenger type or loading further indicates that any scavenger-induced changes in cure kinetics were not large enough to translate into macroscopic differences in panel porosity or accessible hydrophilic sites under the conditions applied.

#### 3.2.3. Thickness Swelling

**Figure 5:**
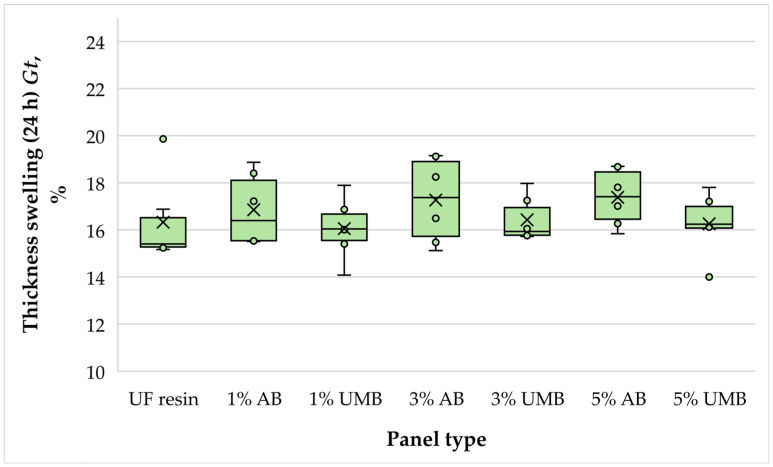
Thickness swelling of the laboratory-fabricated MDF panels bonded with low-molar-ratio UF resin modified with ammonium bisulfite (AB) and urea–metabisulfite (UMB) at 1, 3, and 5 wt%.

The average thickness swelling (TS) values were very similar across all panel series, ranging from approximately 16.05% to 17.39% (Figure 5). The control panels showed a mean TS of 16.33%, and the modified boards exhibited only minor deviations around this level. The lowest mean TS was recorded for panels containing 1 wt% urea–metabisulfite (16.05%), whereas the highest was observed for panels containing 5 wt% ammonium bisulfite (17.39%). However, one-way ANOVA confirmed that these differences were not statistically significant (*p* = 0.599). Therefore, within the investigated concentration range, the addition of either scavenger did not measurably impair the dimensional stability of the MDF panels. This outcome is noteworthy because some studies, particularly on particleboard, have reported increased TS when sulfite-based scavengers were applied at higher dosages, with ammonium bisulfite often associated with a stronger penalty [21,43]. In the present study, the absence of a significant drawback is likely related to the moderate scavenger loadings and the conservative pressing schedule adopted for low-molar-ratio UF-bonded MDF manufacturing. In addition, MDF typically exhibits a more uniform density profile and finer fiber network than particleboard, which can mitigate localized resin starvation and reduce pathways for moisture-driven swelling. The comparable TS values across formulations further suggest that any scavenger-induced shifts in cure development were not sufficient to reduce crosslink density or inter-fiber bonding to a degree that would affect macroscopic thickness recovery after soaking.

#### 3.2.4. Modulus of Elasticity (MOE)

**Figure 6:**
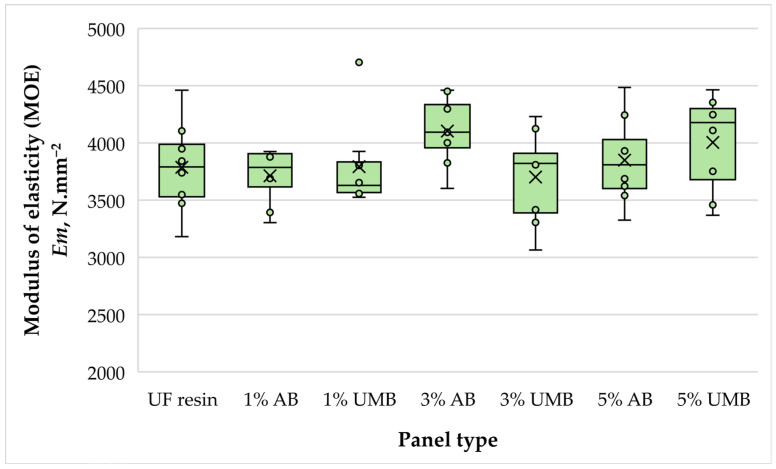
Modulus of elasticity (MOE) of the laboratory-fabricated MDF panels bonded with low-molar-ratio UF resin modified with ammonium bisulfite (AB) and urea–metabisulfite (UMB) at 1, 3, and 5 wt%.

The modulus of elasticity (MOE) remained comparatively high across all panel variants, with group means ranging from approximately 3702 to 4103 N mm^−2^ (Figure 6). The control panels showed an average MOE of 3787 N mm^−2^, while the highest mean value was recorded for panels containing 3 wt% ammonium bisulfite (4103 N mm^−2^). The lowest mean was observed for panels containing 3 wt% urea–metabisulfite (3702 N mm^−2^). Despite these numerical differences, one-way ANOVA indicated that the effect of formulation on MOE was not statistically significant (*p* = 0.261). Thus, within the investigated loading range, scavenger addition did not measurably influence panel stiffness. This suggests that the modified UF systems maintained effective load transfer within the fiber network and that the curing changes detected by thermal analysis did not translate into a loss of flexural rigidity at the board level. In contrast to reports where high scavenger dosages led to pronounced MOE reductions, particularly in particleboard [21,26,43], the present results support the conclusion that the adopted MDF pressing schedule provided sufficient thermal and time reserve to achieve complete or near-complete curing of the modified adhesive systems. Moreover, stiffness in MDF is strongly governed by the integrity of the fiber–resin interphase and the continuity of the cured network; the comparable MOE values therefore imply that interfacial bonding was not compromised by either scavenger at the studied loadings. Taken together with the stable density and swelling behavior, the MOE results indicate that scavenger addition did not introduce structural defects, e.g., insufficient bonding or microcracking, that would normally be reflected in reduced elastic response [2,21,43].

#### 3.2.5. Bending Strength (MOR)

**Figure 7:**
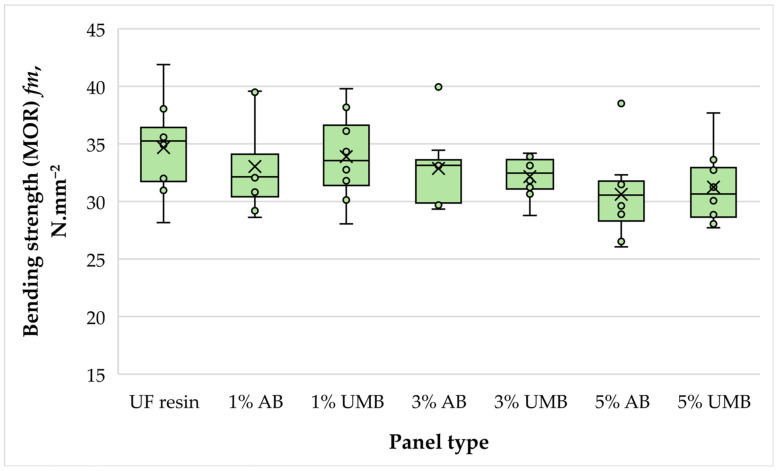
Bending strength (MOR) of the laboratory-fabricated MDF panels bonded with low-molar-ratio UF resin modified with ammonium bisulfite (AB) and urea-–metabisulfite (UMB) at 1, 3, and 5 wt%.

Mean bending strength (MOR) values ranged from approximately 30.6 to 34.7 N mm^−2^ (Figure 7). The control panels exhibited an average MOR of 34.68 N mm^−2^, whereas the modified panels showed only moderate variation around this level. Among the modified groups, the highest mean was recorded for 1 wt% urea–metabisulfite (33.89 N mm^−2^), while the lowest was observed for 5 wt% ammonium bisulfite (30.62 N mm^−2^). However, one-way ANOVA indicated that these differences were not statistically significant (*p* = 0.321). Thus, within the investigated loading range, neither scavenger produced a measurable effect on bending strength. This outcome is technologically relevant because MOR is typically sensitive to insufficient bond development and non-uniform curing through the board thickness. The retention of MOR suggests that the adopted hot-pressing regime (30 s mm^−1^ at 180 °C) provided sufficient curing reserve to accommodate the peak shift or cure broadening identified by thermal analysis. In this respect, the present results are more favorable than those reported in studies where higher scavenger dosages resulted in significant MOR losses [21,26] and are consistent with reports showing that formaldehyde-reducing additives can be applied without major mechanical penalties when dosage and pressing conditions are appropriately balanced [26,44]. The slightly lower mean MOR observed at 5 wt% ammonium bisulfite, although not significant, may indicate the onset of a dosage-related effect and merits attention if higher loadings or shorter pressing schedules are considered. Overall, the combined stability of MOE and MOR supports the interpretation that the scavenger-modified UF systems maintained a sufficiently continuous cured network and adequate inter-fiber bonding to sustain flexural load-bearing performance.

#### 3.2.6. Internal Bond Strength (IB)

**Figure 8:**
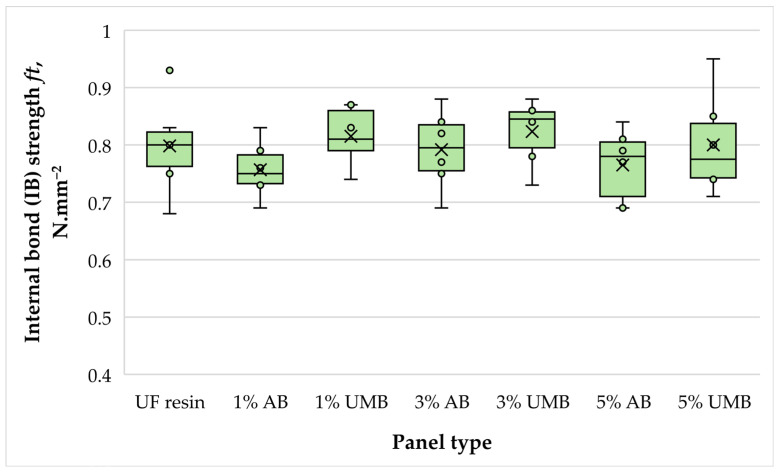
Internal bond strength (IB) of the laboratory-fabricated MDF panels bonded with low-molar-ratio UF resin modified with ammonium bisulfite (AB) and urea–metabisulfite (UMB) at 1, 3, and 5 wt%.

Internal bond strength (IB) values were very similar across all panel series. The control panels exhibited an average IB of 0.798 N mm^−2^, while the modified variants ranged from 0.757 N mm^−2^ (1 wt% ammonium bisulfite) to 0.823 N mm^−2^ (3 wt% urea–metabisulfite) (Figure 8). One-way ANOVA confirmed that these differences were not statistically significant (*p* = 0.581). Because IB is one of the most sensitive indicators of adhesive efficiency and cure completeness in MDF, the absence of a significant reduction supports the conclusion that the scavenger-modified UF systems developed adequate bonding under the selected pressing conditions. This finding is particularly relevant given reports that ammonium bisulfite can reduce IB in particleboard at higher dosages or under less favorable pressing regimes [21,26]; in the present MDF system, no statistically supported penalty was observed within the 1–5 wt% range. The stable IB values are likely attributable to the moderate scavenger loadings combined with the relatively long pressing factor, which favored uniform through-thickness cure development and minimized the risk of under-cured core regions. Together with the unchanged bending properties, the IB results indicate that any scavenger-related modifications of cure kinetics did not translate into a weakening of the core bond line, which is typically the most critical zone for tensile failure in MDF.

#### 3.2.7. General Interpretation of the Property Results

Taken together, the physical and mechanical results indicate that incorporating ammonium bisulfite or urea–metabisulfite up to 5 wt% (based on oven-dry UF resin solids) did not significantly affect density, water absorption, thickness swelling, MOE, MOR, or internal bond strength of the laboratory MDF panels. This is noteworthy because thermal analysis showed that both scavengers modified the curing profile of the low-molar-ratio UF resin. Nevertheless, under the selected hot-pressing conditions, these formulation-dependent thermal differences did not translate into statistically significant changes in board performance. A plausible explanation is that the adopted pressing regime (180 °C and 30 s mm^−1^) provided sufficient thermal and time reserve to accommodate delayed or redistributed curing in the modified systems. As reported in the literature, pressing parameters strongly govern heat transfer, cure development, and final board properties, particularly for fiber-based mats and UF-bonded panels [21,22,25]. Accordingly, the present results suggest that, within the investigated loading range, the influence of the scavengers on physical and mechanical performance was effectively buffered by the manufacturing regime.

Beyond the pressing factor, the absence of property worsening also implies that the scavengers did not induce adverse side effects, such as resin phase separation, poor resin distribution on fibers, or a strong decrease in effective crosslink density that would be reflected in IB, MOR, or swelling. The combined stability of stiffness (MOE) and strength (MOR and IB), together with unchanged moisture-related responses (WA and TS), supports the interpretation that adequate network formation and fiber–resin interfacial bonding were achieved despite the altered thermal profiles. It is also important to note that MDF, due to its fine and relatively homogeneous fiber structure, can be less prone to localized under-cure than particleboard, provided that core heating is sufficient. This may partially explain why effects reported for sulfite-based scavengers in coarser boards are not necessarily reproduced here [21,43]. From an application perspective, these findings indicate that the studied scavenger range (1–5 wt%) is compatible with maintaining baseline MDF performance, but they also suggest that any reduction in press time/temperature or further increases in scavenger loading would require renewed verification of cure completeness and bonding. From a practical perspective, this outcome is advantageous because it allows subsequent evaluation of these additives to focus primarily on formaldehyde reduction efficiency and curing behavior, while maintaining confidence that the principal service-relevant MDF properties can be preserved under comparable pressing conditions.

### 3.3. Formaldehyde Content of the MDF Panels

**Figure 9:**
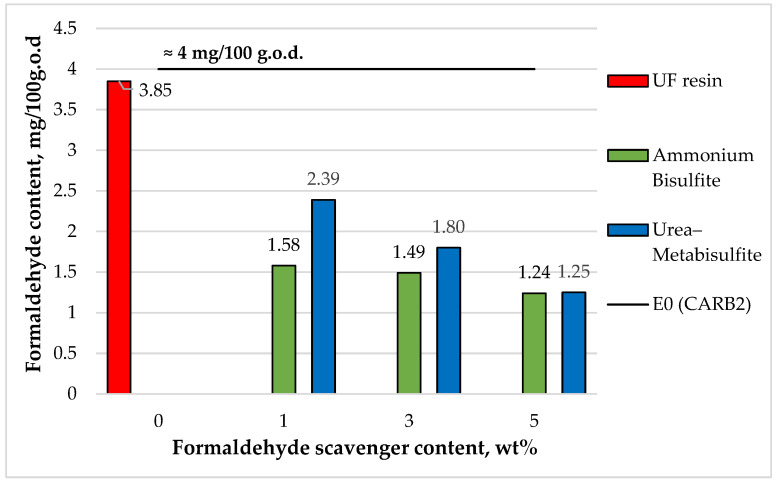
Formaldehyde content of the laboratory-fabricated MDF panels bonded with low-molar-ratio UF resin modified with ammonium bisulfite and urea–metabisulfite, determined by the perforator method (EN ISO 12460-5:2015), as a function of scavenger content.

Perforator testing showed that the low-molar-ratio UF resin already provided a low baseline and that both scavengers further reduced the formaldehyde content in a clear dose-dependent manner (Figure 9). The control MDF exhibited 3.84 mg/100 g (oven-dry board), i.e., below the E0 limit (4 mg/100 g). With ammonium bisulfite, the formaldehyde content dropped sharply already at low loading (e.g., 1.58 mg/100 g at 1 wt%) and reached the 1.36–1.24 mg/100 g range at higher additions (Figure 8), corresponding to an overall reduction of roughly ~59–68% relative to the control. Urea–metabisulfite also produced a progressive decrease, from 2.40 mg/100 g at 1 wt% to 1.26 mg/100 g at 5 wt% (≈67% reduction). Thus, while both scavengers were effective, ammonium bisulfite delivered a stronger reduction at low-to-intermediate dosages, whereas urea–metabisulfite required higher loading to reach comparable levels.

From an application perspective, the most relevant outcome is the dosage required to reach the super E0 target (<1.5 mg/100 g). This threshold was achieved at 3 wt% ammonium bisulfite, while urea–metabisulfite reached it only at 5 wt% (Figure 8). This difference is relevant from an application standpoint because it indicates that the two scavengers differ not only in their impact on curing behavior, but also in the dosage required to achieve ultra-low formaldehyde levels in the final MDF panels.

A plausible explanation for the higher dosage efficiency of ammonium bisulfite is its rapid bisulfite/sulfite-type addition to free formaldehyde, forming hydroxymethanesulfonate-type adducts and thereby depleting the unbound formaldehyde pool more effectively at low dosages [44,45]. In contrast, urea–metabisulfite likely acts through a more complex interplay of formaldehyde capture, shifts in resin equilibria, and modification of cure development. This interpretation is consistent with the thermal analysis observations: ammonium bisulfite mainly shifted the principal curing peak, whereas urea–metabisulfite produced a broader, multi-step exotherm, suggesting a scavenging effect distributed over a wider temperature interval and becoming competitive primarily at higher addition levels.

The present trends align with the literature reporting substantial reductions in perforator values for UF-bonded wood-based panels using sulfite-based scavengers [21,22,26]. Costa et al. observed marked reductions with ammonium bisulfite in particleboards, although higher dosages were associated with penalties in internal bond strength and thickness swelling [3,25]. In this context, the current MDF results are favorable because the pronounced perforator reductions were achieved without statistically significant losses in density, moisture-related properties, or strength-related metrics within the investigated loading range, indicating that the chosen pressing schedule provided sufficient curing reserve for the modified systems.

Finally, it should be emphasized that the perforator method quantifies formaldehyde content (emittable potential) rather than real-time emission under service conditions [21,24]. Because scavenger efficiency may differ among perforator, desiccator, gas analysis, and chamber methods due to differences in temperature, humidity, and air exchange that influence adduct stability and release kinetics [21,24,25], the perforator test is best interpreted here as a standardized comparative framework. Within this framework, ammonium bisulfite is the more dosage-efficient option at moderate addition levels, whereas urea–metabisulfite requires higher loading to reach the same class, and, given the stable panel properties, selection can be guided primarily by formaldehyde reduction efficiency, associated curing behavior, and process robustness under the adopted pressing conditions.

## 4. Conclusions

This study comparatively evaluated ammonium bisulfite and urea–metabisulfite as formaldehyde scavengers in a low-molar-ratio UF resin (F/U = 1.06) for manufacturing ultra-low-formaldehyde MDF panels. A key contribution is the combined assessment of resin-level curing behavior (via simultaneous thermal analysis) and board-level performance, enabling an integrated evaluation of cure development, formaldehyde reduction, and panel properties.

Thermal screening demonstrated that the two scavengers altered the curing profile of the low-molar-ratio UF resin in distinct ways. Ammonium bisulfite shifted the principal exothermic curing event toward higher temperatures, indicating retardation of the dominant polycondensation stage, whereas urea–metabisulfite generated a broader, multi-peak curing profile consistent with a more distributed cure development.

Despite their different curing signatures, both scavengers effectively reduced the perforator formaldehyde content of the laboratory-fabricated MDF panels. The control panels already satisfied the E0 class, while 3 wt% ammonium bisulfite and 5 wt% urea–metabisulfite achieved super E0 levels (<1.5 mg/100 g oven-dry board). Importantly, scavenger additions up to 5 wt% did not cause statistically significant changes in density, water absorption, thickness swelling, MOE, MOR, or internal bond strength, indicating that the selected pressing regime provided sufficient curing reserve to accommodate the modified curing profiles while preserving the main service-relevant panel properties.

From an application perspective, these results suggest that ammonium bisulfite, particularly at 3 wt%, is a practical option for producing super E0 MDF with low-molar-ratio UF resins, provided that adequate curing reserve is ensured in the pressing schedule. Further validation under alternative industrial processing conditions and complementary emission evaluation methods may be addressed in subsequent work.

## Figures and Tables

**Table 1:** Thermal parameters of the control and modified UF adhesive systems.

Adhesive System	Scavenger Content (wt%)	T_onset_ (°C)	T_peak_ (°C)	T_end_ (°C)	Curing Interval (°C)	Normalized Peak Heat Flow (mW g^−1^)	Apparent Thermal Effect (J g^−1^)	Interpretation
UF control	0	90.6	124.4	194.7	104.1	−153.4	76.8	Single dominant exotherm
UF plus ammonium bisulfite	1	86.8	126.1	200.1	113.3	−171.3	96.3	Slight shift to higher temperature
UF plus ammonium bisulfite	3	80.9	139.5	196.0	115.0	−318.8	131.6	Strongly delayed but concentrated cure
UF plus ammonium bisulfite	5	80.0	154.5	189.1	109.1	−163.5	93.1	Maximum shift of principal cure peak
UF plus urea–metabisulfite	1	85.3	141.8	183.9	98.6	−144.3	71.7	Main peak shifted upward; still relatively compact
UF plus urea–metabisulfite	3	85.4	107.8	186.5	101.1	−99.6	64.1	Multi-peak/redistributed cure
UF plus urea–metabisulfite	5	74.0	108.9	184.4	110.4	−100.0	54.6	Multi-peak/distributed cure

Note: The heat-flow signal was baseline-corrected and normalized to sample mass. Since the measurements were performed in air and in open crucibles, the integrated thermal effect was treated as an apparent comparative parameter rather than as an absolute curing enthalpy.

## Data Availability

The original contributions presented in this study are included in the article. Further inquiries can be directed to the corresponding authors.
